# Advances in nanotechnology and antibacterial properties of biodegradable food packaging materials

**DOI:** 10.1039/d0ra02922j

**Published:** 2020-05-28

**Authors:** Heba Mohamed Fahmy, Rana Essam Salah Eldin, Esraa Samy Abu Serea, Nourhan Mamdouh Gomaa, Gehad M. AboElmagd, Suzan A. Salem, Ziad A. Elsayed, Aisha Edrees, Engy Shams-Eldin, Ahmed Esmail Shalan

**Affiliations:** Biophysics Department, Faculty of Science, Cairo University 12613 Egypt hfahmy@sci.cu.edu.eg; Chemistry & Zoology Department, Faculty of Science, Cairo University 12613 Egypt; Chemistry & Biochemistry Department, Faculty of Science, Cairo University 12613 Egypt; Physics Department, Faculty of Science, El-Menoufia University Menoufia Egypt; Biophysics Department, Faculty of Women for Arts, Science and Education, Ain Shams University Egypt; Chemistry & Physics Department, Faculty of Science, Cairo University 12613 Egypt; Special Food and Nutrition Department, Food Technology Research Institute, Agriculture Research Center Giza Egypt engi_maged@yahoo.com; Central Metallurgical Research and Development Institute (CMRDI) P.O. Box 87, Helwan Cairo 11421 Egypt; BCMaterials-Basque Center for Materials, Applications and Nanostructures Martina Casiano, UPV/EHU Science Park, Barrio Sarriena s/n Leioa 48940 Spain ahmed.shalan@bcmaterials.net a.shalan133@gmail.com

## Abstract

A large number of non-biodegradable and non-renewable materials are produced daily for application as food packaging materials. These waste materials have a greatly negative effect on our health and the ecosystem. The idea of a bio-based economy is steadily gaining attention from the scientific, societal, and financial communities, so there are several areas in which the intended approaches can be improved for this reason. Therefore, creating biopolymer-based materials from natural sources, including polysaccharides and proteins, is a good alternative to non-renewable fossil resources. In the current review paper, we plan to summarize the major recent findings in food biodegradable packaging materials that include nanotechnology either directly or indirectly. Several natural nano-materials applied in food packaging applications such as polymers, polysaccharides, and protein-based nano-materials have been included in order to make special biopolymer hosts for nanocomposites. Finally, this review will highlight the antibacterial properties of commonly used nanoparticles or nanomaterials.

## Abstract 2

Herein, we aim to summarize the major recent findings in food biodegradable packaging materials that include nanotechnology either directly or indirectly.

## Graphical abstract

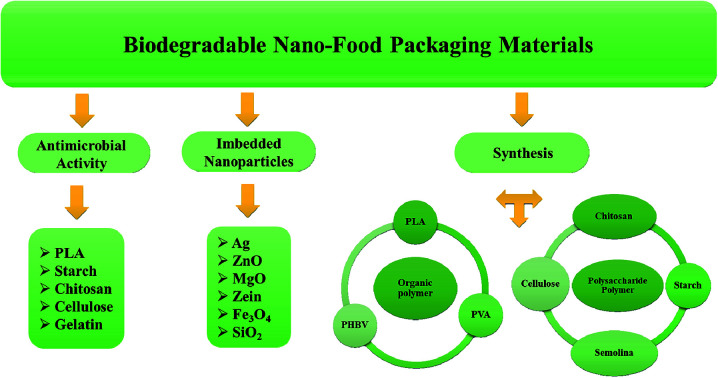

## 1 Introduction

Nowadays, a large number of non-biodegradable and non-renewable materials are produced daily for “only one use” in the food packaging field. These waste materials such as glass, metals, and plastics contaminate our environment. Furthermore, huge amounts of these wastes are treated through land-filling or even burning, which has a greatly negative effect on our health and the ecosystem. Hence, eco-friendly products have attracted wide interest as safe and non-toxic packaging materials. Furthermore, the limitations of petroleum resources have pushed the country to focus on different resources such as forest and agricultural origins.^1^ Different communities including scientific, social, and financial ones have given importance to a bio-based economy due to its thrust towards a clean environment.^2^ Creating biopolymer-based materials from natural sources, including polysaccharides and proteins, is a good alternative to non-renewable fossil sources. In addition, there are different natural nano-materials applied in food packaging applications such as polymer-based plastics, and polysaccharide-based and protein-based nano-materials that make special biopolymer hosts for nanocomposite materials.^3^

It is very promising that nanotechnology is used in food packaging because this technique could enhance food protection and superiority while decreasing the use of precious raw materials and waste generation. Nanomaterials are defined as insoluble materials with an inner composition in the range from 1 to 100 nm.^4^ Most new features of food packaging products will probably include nanomaterial-based solutions either directly or indirectly because they are necessary for improving gas-barrier, antimicrobial, mechanical, and thermal features, which are essential for packaging materials.^5^

The utilization of different particles that depend on metals, oxides, and organic compounds is vital to enhance the polymer antimicrobial activity. For example, silver (Ag) particles can be used for this purpose because Ag shows antibacterial and antifungal capacity against several types of bacteria (around 150) and it is already found in different commercial products.^6^

On the other hand, although the cost of polylactic acid (PLA) is high compared to polyolefins such as polypropylene (PP) and polyethylene(PE), there is an abundant awareness towards using PLA based-materials in food packaging as alternative films from compostable and biodegradable natural sources.^6^ In the current review, we will explore the recent advances in nanotechnology, safety, as well as the antibacterial properties of the materials utilized in biodegradable food packaging.

## 2 Biodegradable nano-food packaging materials

There are several eco-friendly approaches for the preparation of biodegradable/biocomposite nanomaterials designed for food packaging applications such as microwave, ultrasound, and electrospinning methods. Microwave (MW) is considered as one of the practical techniques to obtain nanomaterials *via* green synthetic pathways. In addition, the heating technique in the MW method is considered as advantageous as it has shorter reaction periods, small energy depletion, and enhanced product yields that inhibits the agglomeration of the obtained particles. Furthermore, different structures of nanoparticles, including spherical, single crystalline polygonal plates, sheets, rods, wires, tubes, and dendrites can be obtained within a few minutes under MW heating conditions and other parameters such as the concentration of metallic precursors, surfactant polymers, solvents, operational temperature, morphology, and nanostructure size can be controlled.^7,8^ Subsequently, the chemical effects of ultra-sonication, wherein sound energy is applied to physical and chemical systems, result from hot spots that are attained in the course of acoustic cavitation by the progress and collapse of bubbles in a liquid.^9,10^ Many studies have been carried out to focus on the important effects of ultra-sonication in the degradation of polysaccharide linkages.^11^ On the other hand, another possible technique that is considered as an eco-friendly approach for the preparation of biodegradable/biocomposite nanomaterials for food packaging applications is the “electrospinning method”, which is based on spinning of fibers with diameters of >100 nm up to the micrometer level in order to obtain a wide range of polymers. This electrostatic treatment utilizes a high-voltage electric field in order to attain solid fibers from a polymeric fluid stream (solution or melt) provided side to side with a millimeter-scale nozzle.^12^ Polymers have a promising future in several applications; one of the most important is food packaging. Moreover, reviewing the different characterization techniques used for studying the alteration in several obtained polymer materials (homogeneous and heterogeneous polymer systems) in the presence of packaging gases and in dissimilar environmental situations is essential to recognize the adaptation of the carefully chosen material in the food packaging field.^13^ Polymers are characterized by their balance between physical and chemical properties; in addition, they are easy to process. There are some properties that should be possessed by polymeric materials to enable their use in food packaging applications such as barrier properties that allow the exchange of low molecular weight materials at a higher or lower level through permeation, absorption, and migration processes. However, the migration of some low molecular weight materials from plastic packaging walls may cause damage or degradation of the quality of products; therefore, the transfer of low molecular weight substances through polymers is not appropriate in food packaging applications. Moreover, the mass transport property is considered as one of the positive properties of the polymers in the case of intended migration, which is useful in the application of anti-microbial polymers and the techniques of active packaging. Collectively, some polymers are subjected to some chemical modifications in order to obtain the anti-microbial activity but these modifications should not affect the final polymer properties.^14,15^

### 2.1 Organic polymer nanocomposites

**Fig. 1:**
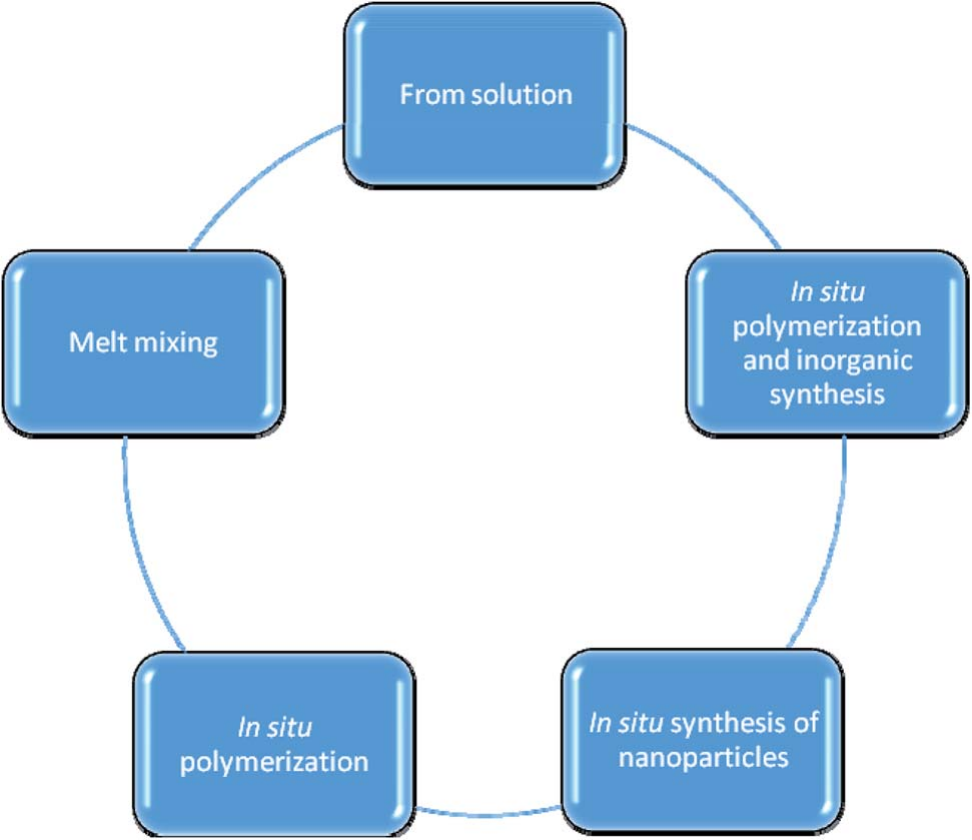
The schematic diagram summarizes the different approaches of polymer nanocomposites production.

Generally, polystyrene (PS) and polyethylene (PE), as petroleum-based polymers, were used as plastic packaging materials in different cases but these materials are considered as harmful with respect to the environment. On the other hand, novel polymer materials (biodegradable polymer-based plastics) are unconventional choices, for example, polylactic acid (PLA),^16^ polyvinyl acetate (PVA),^17^ poly(3-hydroxybutyrate-*co*-3-hydroxyvalerate) (PHBV),^18^ and their biopolymer mixes. The high surface-to-volume ratio can affect the performance of the obtained materials as it can considerably adapt the intrinsic features of a polymer matrix with very low incorporated quantity. Two primary mechanisms for the production of polymer nanocomposites are used, namely, the *in situ* synthesis of inorganic particles (*e.g.*, metal oxides) as well as the addition of fillers (*e.g.* layered nanoclays), which can be produced in a polymerized matrix (Fig. 1).^5^

• Polymer preparation from solution

The exfoliation adsorption method^5^ involves the addition of a dispersed pre-polymer into a solution that contains layers of silicate. Usually, this methodology employs water soluble polymers of low polarity, for example, poly(vinyl alcohol), poly(ethylene oxide), poly(vinyl pyrrolidone), and poly(acrylic acid), to create intercalated nanocomposites.^19^ However, this method has disadvantageous because it is not eco-friendly.^5^

• Polymer/nanoparticle composite preparation *via* the melt mixing method

In this, the polymer and nanoparticles are mixed under a specific pressure and temperature (greater than the softening point of the polymer).^20^ Often, this approach is used to produce layered polymer nanocomposites.^21^ Moreover, the polarity variance between the organic polymer and the inorganic nanomaterial can seriously prevent its distribution and reduce the development of interaction.^5–9^ Therefore, several factors can negatively affect the final product (*e.g.*, temperature, pressure, and the functional groups on the nanoparticles).^22^ The melt mixing method is extensively used due to its safety with respect to the environment.^5^

• Polymer preparation *via in situ* polymerization

*In situ* polymerization is carried out by filler swelling in a monomer solution. There are some processes that initiate the polymerization process such as the application of heat and radiation, which induce the subsequent polyaddition of monomers to give the final nanomaterials.^23^ This method has an advantage over the melt mixing and exfoliation methods due to the improvement in the exfoliation.^5^

• Polymer preparation *via* the *in situ* synthesis of nanocomposites

In this method, a polymer acts like a reaction medium, wherein inorganic nanoparticles are formed because the organic polymer is considered to be a stable chemical material. Subsequently, the resultant nanocomposites have synergetic effects and can be used in new applications that are not available with the polymer or the nanoparticles alone.^5^ Metal or metal oxide particles are produced by means of a metal precursor in the polymer phase. The *in situ* method permits the adjustment of the obtained particle size as well as the morphology.^5–14^

• Inorganic synthesis of polymer *via in situ* polymerization

The sol–gel technique is considered as the most widely utilized process towards the *in situ* processing of polymer nanocomposites. This technique is associated with two responses that contribute to the shift from the solution state to the gel state, *i.e.*, the colloidal suspension of strong ions in the liquid state.^5^

#### 2.1.1 Polylactic acid (PLA)

The great biodegradability of PLA materials makes them more favorable as promising replacements of conventional polymers. In addition, PLA is formed using the condensation polymerization of lactic acid, which is produced *via* the fermentation of corn, sugars, and sugarcane. Furthermore, among other biopolymers, PLA is favored because of its biodegradability, renewability, and good mechanical features.^24^

In addition, PLA has great mechanical properties as the principal bio-based plastic created at a large scale, mostly by hydrolytic degradation.^25^ Many scientific studies^26^ have considered the effect of calcium carbonate (CaCO_3_) nanoparticles towards PLA biodegradability and its obstructing features. It was additionally discovered that the absorptivity of CO_2_, O_2_, and N_2_ gases was improved by raising the temperature; however, it was reduced on increasing the pressure. In addition, the Cu-doped ZnO powder was incorporated inside the PLA biopolymer structure in a previous study.^27^ The desired materials were functionalized with Ag-NPs *via* the melt blending method. The outcomes demonstrated a gradual increase in the crystallinity of PLA on increasing the mixing amount of the nanoparticles in the range of 0.5–1.5%. In spite of the fact that the creation innovation of PLA has been enormously developed, there are still numerous directions in which the applications of PLA can be developed. Nowadays, PLA cannot completely replace the traditional thermoplastic materials. In addition, the applications are also restricted to certain conditions due to the fragility of PLA. At last, when PLA is exposed to different climatic conditions, it might show unknown behavior. Accordingly, in any case, the utilization of polylactic acid could be broad if its performance is enhanced. Of late, scientists have utilized a variety of nanofillers to improve the performance of PLA.^28^ The great compatibility, material quality, and low cost of PLA make it broadly utilized in medical applications.^29^

Even though PLA shows important commercial potential, some natural properties should be improved such as the low temperature of heat distortion, low melt viscosity, and weakness. All these problems restrict its use. In addition, antibacterial, barrier, and mechanical properties are of special importance in food packing applications.^6^

• PLA/oleic acid-TiO_2_

A previous study mentioned the improvement in the properties of polylactic acid (PLA) film due to the addition of stabilized TiO_2_.^30^ Three forms of PLA were prepared *via* solvent casting pathway, investigated, and compared. These were named as PLA as a reference, PLA with oleic acid-improved TiO_2_ (OT-PLA), and PLA with original TiO_2_ (T-PLA). The OT-PLA film had advantages over the PLA film such as better UV blocking, and oxygen (O_2_) and water barriers. The uniform distribution of stabilized TiO_2_ in the matrices of PLA prevented the presence of clusters and agglomerations. Moreover, the mechanical properties showed that OT-PLA has higher elasticity and lower Young's modulus than T-PLA and PLA. Compared with PLA, the oxygen absorptivity and water vapor of 1% OT-PLA were decreased by 29% and 26%, respectively. Furthermore, TiO_2_ protects the film from UV-light degradation.^31^ Collectively, PLA with oleic acid-modified TiO_2_ can be utilized in food packaging for the reason that the amalgamation of PLA with modified TiO_2_ increased the flexibility and decreased the moistness in addition to being an eco-friendly material.^30^

• PLA/lignin/silver nanocomposite film

Furthermore, the same study used organosolv lignin to produce the composite film, by means of a reduction agent, to synthesis and incorporate silver nanoparticles (AgNPs) inside the PLA polymer.^30^ The findings of Fourier transform infrared (FTIR) spectroscopy showed that once lignin and AgNP were incorporated, the chemical structure of PLA was not changed. The PLA film was extremely transparent as no light above 240 nm was absorbed and the transmission of light at 280 nm reduced significantly after the combination of lignin and AgNP. Phenolic and carbonyl groups in lignin absorb light near the UV range.^32^ The PLA/lignin/AgNP composite film was smooth and flexible. It was observed that the mechanical properties were increased while the elasticity of the film did not change and the water vapor permeability was decreased. The AgNPs are active against food-borne pathogenic bacteria (*Escherichia coli* and *Listeria monocytogenes*).^33^

• PLA/zinc oxide nanocomposite film

The antibacterial activities, barriers, and mechanical characteristics are especially interesting for the implementation of food packaging. Various metal oxides such as zinc oxide (ZnO), titanium dioxide (TiO_2_), magnesium oxide (MgO), and silicon dioxide (SiO_2_) are popular owing to their capacity in preventing UV radiation as well as antibacterial agents. For food packaging materials, MgO and ZnO particles are the principal and safest nanoparticles aimed at that purpose.^6^

**Fig. 2:**
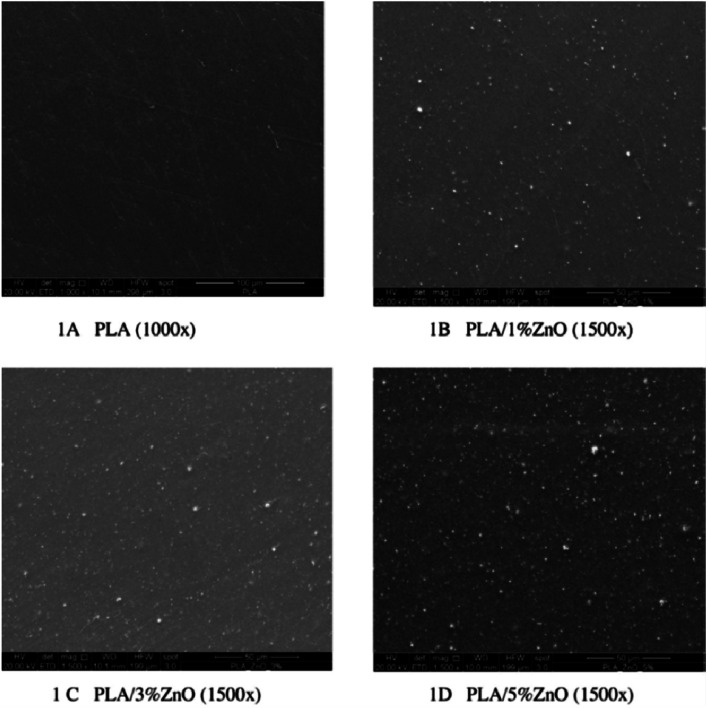
The scanning electron micrographs of the surfaces of (A) PLA and (B–D) the PLA/ZnO bio-composite films with different addition percentage (1, 3, and 5%), respectively. The scale bar for (a) is 100 μm, while it is 50 μm for (b–d). Reproduced with permission from ref. 6. Copyright© 2016 Elsevier.

Fig. 2 illustrates the SEM images of the obtained PLA as well as the doped materials with different compositions of ZnO nanoparticles (1, 3, and 5%) in the doping percentage. The results show the homogeneous distribution of ZnO agglomerates on the surface of the film. Moreover, adding ZnO to PLA strengthens the obtained film. The amount of permeability for oxygen (O_2_), carbon dioxide (H_2_O), and water vapor was detected. The addition of 1% (w/w) of ZnO decreased the O_2_ permeability by about 18%. On the other hand, the addition of ZnO at the same ratio decreased the CO_2_ permeability by about 17% compared to PLA. But the further addition of ZnO for both the other films did not induce any more reduction in the permeability of O_2_ and CO_2_ gases. In addition, the ZnO nanoparticles had antibacterial capacity against bacteria, which was not affected by high temperature and pressure, depending on the surface area and concentration of the nanoparticles.^6^

#### 2.1.2 Polyvinyl acetate (PVA)

PVA, essentially produced using polyvinyl acetate hydrolysis, is degraded easily by organisms in water. Furthermore, several polymers were combined with it and were mixed for different reasons such as improving the mechanical properties (according to its perfect structure), solvent resistance, biocompatibility, as well as increasing the hydrophilicity.^34^ In addition, chitosan/PVA hydrogels with lignin nanoparticles (LNPs) were prepared in two ratios, *i.e.*, 1% and 3%, *via* the freezing–thaw method.^17^ Furthermore, the investigation of mechanical, microstructural, and thermal characteristics of the produced material demonstrated that the best one of the LNPs was obtained at 1%, while the agglomerates were formed at higher LNP content and negatively affected the properties.^35^ In addition, recent studies explored the effect of Ag nanoparticles, which were inserted inside nanocellulose for improving the mechanical, physical, and thermal properties of the obtained PVA-based nanocomposite films.^36^ The results indicate the predominant antimicrobial action of the obtained films against *E. coli* and *S. aureus*. In contrast, the films revealed no cytotoxic impact against HepG2.

#### 2.1.3 Poly(3-hydroxybutyrate-*co*-3-hydroxyvalerate), PHBV

One of the most important biodegradable and biocompatible thermoplastic food packaging materials are polyhydroxyl alkanoates (PHAs), which have gained more attention from researchers.^18^ Furthermore, poly(3-hydroxybutyrate), PHB, is one of the essential examined polymers from the PHAs, which is incompletely crystalline with extraordinary inflexibility and a high melting temperature. In order to reduce the crystallinity, the copolymer produced *via* embedded 3-hydroxyvalerate (HV) monomer to produce poly(3-hydroxybutyrate-*co*-3-hydroxyvalerate) (PHBV) with developed characteristic properties of the PHB films.^18^ Several studies concerning the synthesis of PLA and PHBV mixes at various weight proportions ranged from zero to one for the individual polymer, using the compounding melt process.^37^ In addition, the results indicated that improving the amount of PLA inside the PLA/PHBV mixture can enhance the flammability resistance and thermal stability of the prepared materials.^37^

• PHBV/silver

A new PHBV material with *in situ* stabilized silver nanocomposite was successful synthesized and characterized.^38^ Nanoparticles such as silver may be utilized for the improvement of food packaging materials to obtain environment friendly and biodegradable packaging materials, which have industrial importance depending on consumer needs.^38^ However, the incorporation of Ag (at concentration 0.04% (w/w)) into PHBV led to a decrease in the O_2_ permeability to about 56% with respect to the neat polymer, while the optical properties and thermal stability did not change. Moreover, the antibacterial activity of the new film was assessed against two of the strongest food borne pathogens (*Salmonella enterica* and *Listeria monocytogenes*), where the film exhibited exceptional antibacterial food contact activity for seven months. This promising film was formed from biodegradable materials from food manufacturing by-products *via* the melt blending method that allows the addition of low stabilized AgNPs concentration without any need for further additives.

**Fig. 3:**
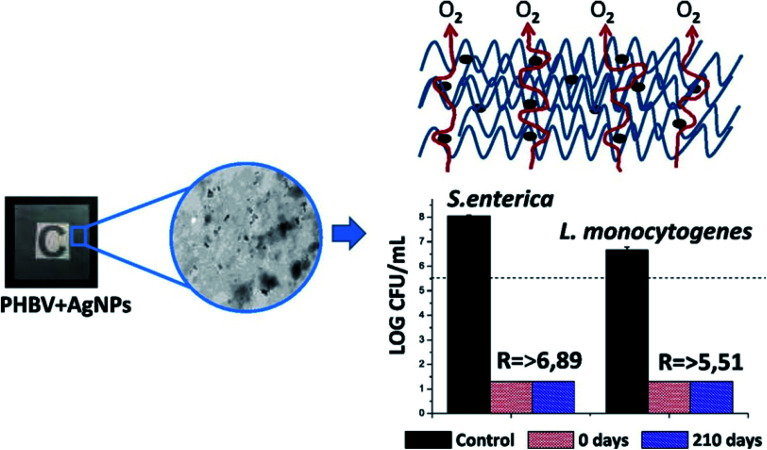
The figure shows firstly, the incorporation of AgNPs into the PHBV structure, then the evolution of O_2_ gas during the formation process. After that, it shows the antimicrobial activities of freshly made nanocomposites (0 days) and 210 days aged PHBV3/PHBV18/AgNPs nanocomposites against *Salmonella enterica* and *Listeria monocytogenes* after 24 h exposure. The dashed line depicts the initial inoculum size of 5.6 log CFU. The detection limit was 20 CFU mL^−1^. Reproduced with permission from ref. 38. Copyright© 2015 Elsevier.

Good dispersion as well as compatibility of the PHBV18/AgNPs and PHBV3 matrices were observed.^38^ Furthermore, thermal degradation and the presence of yellowish color of this film did not happen due to the presence of AgNPs, in which the thermal stability and transparency are considered as an important factor in the food packaging industry. Silver nanoparticles have a biocidal influence at an extremely low concentration (0.040 ± 0.002%), which was estimated by ICP-OES. Fig. 3 represents that the film with silver nanocomposites revealed a decrease of about 6.89 log CFU in case of *Salmonella enteric*, while the decrease reached up to 5.51 log CFU in the case of *Listeria monocytogenes*, as compared to PHBV3/PHBV18 (without silver nanoparticles).^38^

• PHBV/ZnO

**Fig. 4:**
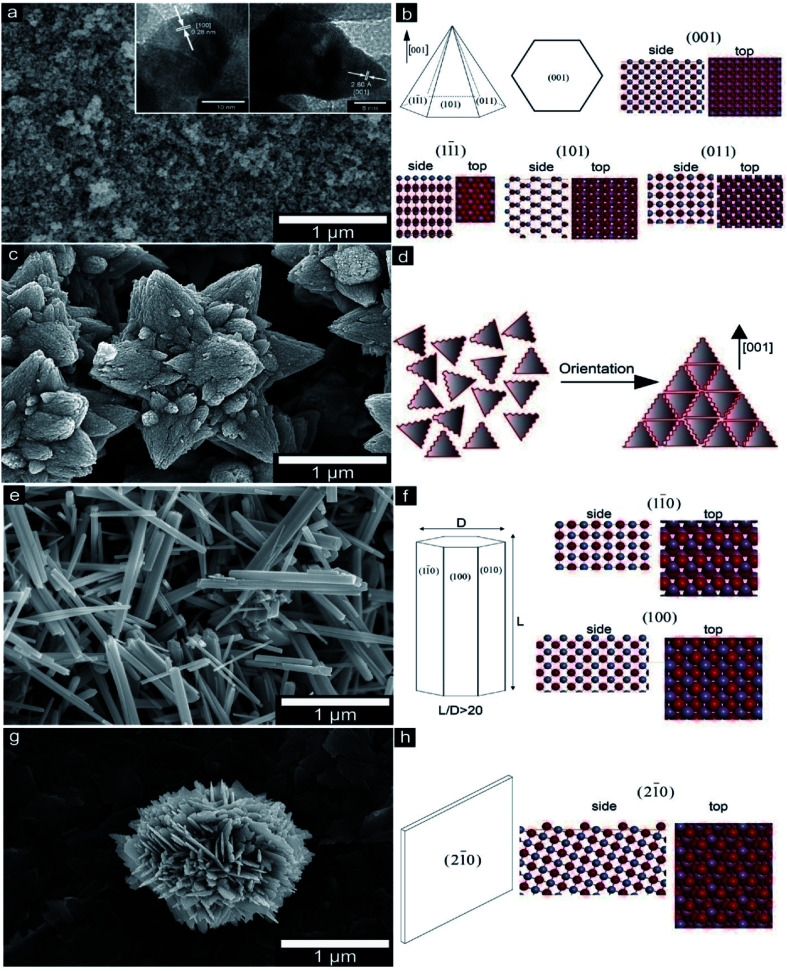
The SEM micrographs of ZnO particles and the crystal model of different planar terminations. (a and b) P–ZnO, (c and d) S–Zn, (e and f) R–ZnO, (g and h) B–ZnO. Reproduced with permission from ref. 39 Copyright© 2016 Elsevier.

**Fig. 5:**
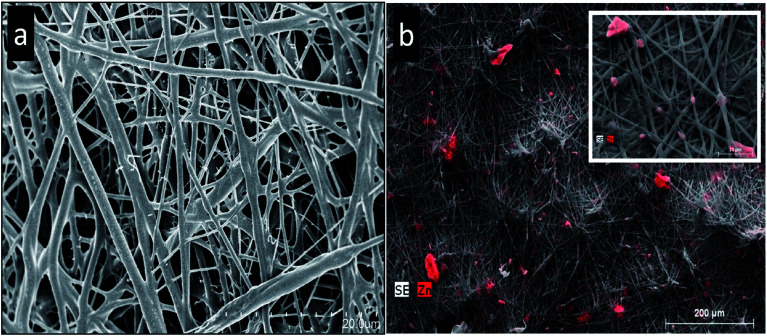
The SEM micrographs of electrospun PHBV18: (a) PHBV18 without ZnO. (b) PHBV18 with P–ZnO incorporated by electrospinning. The elemental map analysis for zinc was carried out using EDAX on the SEM micrographs; the results of mapping are shown in red. The inset shows the detailed image of the fibers containing P–ZnO. Reproduced with permission from ref. 39 Copyright© 2016 Elsevier.

A recent work investigated the various morphological effects of ZnO as well as the crystal model of several planar terminations (Fig. 4) in micron as well as ZnO nanosized particles when incorporated into the PHBV film (Fig. 5), which was synthesized *via* water precipitation.^39^ There was a directly proportional relationship between the antibacterial properties and the exposed surface area of the different ZnO nanoparticles (hexagonal-pyramid nanoparticles represented the higher antibacterial effect). On the other hand, the incorporation of ZnO nanoparticles developed the thermal stability as well as the optical features of the films that can prevent the conversion of color into brown after thermal processing.

Although literature has stated that ZnO nanoparticles enriched the mechanical and the barrier features when incorporated in biopolymeric matrices, large amounts of ZnO, which is needed for the antibacterial activity (*Listeria monocytogenes*), negatively affected the properties of the host material.^39^ It might be due to the lower crystallinity and the greater hydrophilic character of ZnO than the neat PHBV.^40^ In addition, the significant dispersion, in addition to the distribution of ZnO accomplished by the electrospinning pre-incorporation process, limits this negative effect on the barrier features.

### 2.2 Polymer nanocomposites of polysaccharide

Nanocomposite production shows promising prospective in research and application directions, as the utilization of particles in the linear dimension lower than 100 nm will pave the way towards enhancing the investments in this industry. In addition, most of the nanocomposite structures can be obtained *via* different polymer structures such as polysaccharides (cellulose, starch, and chitin), depending on the hierarchical structure and the semi-crystalline nature of these polymers. Furthermore, polysaccharide nanocrystals retain the sensitive surface exposed by the hydroxyl groups, thus showing the probability to modify widespread chemical reactions. Subsequently, polysaccharide nanoparticles are achieved in the aqueous suspension phase and most research studies are dedicated to hydro-soluble (or at least hydro-dispersible) or latex-form polymers. On the other hand, not only is the aqueous phase the possible phase of the materials but also, the existence of surfactants or chemical grafting can disperse these materials in non-aqueous media and it can pave the way towards other prospects for the synthesis of nanocomposite materials.

### 2.3 Biodegradability of the polymers after nanocomposite/composite formation

The improvement of novel nanocomposites depends on the features and several conditions of biodegradation, such as hydrolytic, composting, and enzymatic properties intended for biodegradable polymers, which put the bio-based nanocomposites and biodegradable nanocomposites at the top of the research interest related to the essential point of food packaging pathway. Furthermore, polymer nanocomposites based on commercial polymer matrices have been established and nano-scale filler distribution has been accomplished by changing the points of achievement to attain favorable bio-based and biodegradable polymer matrices incorporated with several nanoscale materials with enhanced properties. These structures comprise biopolymers from agricultural resources such as polysaccharides and proteins, from biotechnology (as an example, poly(lactic acid), poly(hydroxyalkanoates), or biopolymers from petrochemical sources, *e.g.*, PCL). The current section is concerned with the role of polymeric matrices and gives detailed studies regarding the biodegradability effect towards the synthesized nanocomposite/composite materials for food packaging.

#### 2.3.1 Chitosan

Chitosan is consider as a linear polysaccharide formed of β-(1-4)-linked d-glucosamine and *N*-acetyl-d-glucosamine, and it has a lot of useful properties such as biocompatibility, biodegradability, and metal complexation.^3^ The chitosan nanoparticles were formed *via* ionic gelation, by the electrostatic interaction of the amino group of chitosan (positively charged) with the polyanions of other amino acids, thus producing a cross-link.^41^ Earlier, it had been observed that the attraction between both chitin whiskers and soy protein isolate (PI) thermoplastics dramatically promoted the tensile appearance of the matrix and enhanced their resistance to water.^42^ After that, it was suggested that hydroxypropyl methylcellulose (HPMC) can work as a potential substance in packaging films that are not harmful to humans.^43^ De Moura *et al.* discussed that the nanocomposites of chitosan in HPMC helped in enhancing the mechanical and barrier characteristics.^44^ In addition, a previous study related to the formation of chitosan films included the nanocapsules of epigallocatechin gallate antioxidant.^45^ The experimental work indicates that the ratio of the film elongation at break to the lightness was reduced by the addition of nanocapsules into the chitosan films and the process can enhance the tensile strength of the formed compounds.^45^

• Chitosan/TiO_2_ composite film

The obtained composite chitosan/TiO_2_ film was considered to be a successful material aimed at food packaging, where it is effective against pathogenic microbes. The results were positive when the chitosan/TiO_2_ film was used in the packaging of red grapes.^46^

**Fig. 6:**
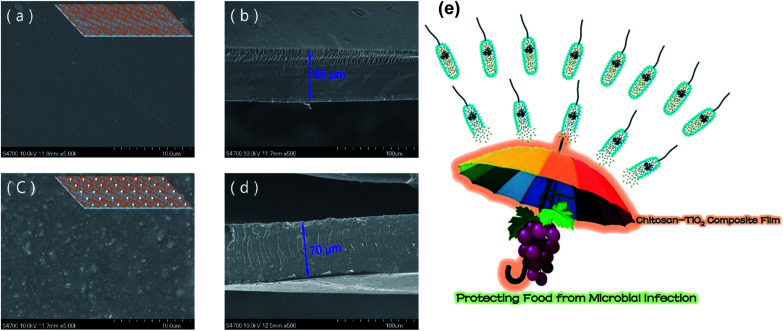
(a) The SEM image of the surface of pure chitosan film with the inserted schematic diagram; (b) the SEM cross-sectional image of pure chitosan film; (c) the SEM surface image of the chitosan/TiO_2_ film with the inserted schematic diagram; (d) the SEM cross-sectional image of the chitosan/TiO_2_ film; (e) the schematic diagram showing the effect of the chitosan–TiO_2_ composite film in protecting food from microbial infection. Reproduced with permission from ref. 46 Copyright© 2017 Elsevier.

This study shows excellent compatibility for the addition of chitosan to TiO_2_. The surface and cross-sectional images of the films presented in Fig. 6 show the surface of the chitosan/TiO_2_ film, which was rough to a certain extent compared to the chitosan film; it could be as a result of the additional TiO_2_ nanoparticles.^47^ However, the structure was as compact as the pure chitosan film with a homogeneous cross-section. The addition of TiO_2_ led to an increase in the TS (up to 89.64%) and *E* (up to 69.21%). This increase was due to the new crystal structure, which formed as a result of the TiO_2_ nanoparticles.^47^ The chitosan/TiO_2_ film reduced the transmission of light due to the existence of TiO_2_ nanoparticles that scatter light and it is shown clearly in Fig. 6e as to how the obtained film can protect food from microbial infection. In addition, four typical food-borne pathogenic microbes were inhibited by the prepared film, namely, *E. coli* (Gram-negative bacteria), *S. aureus* (Gram-positive bacteria), *C. albicans* (fungi), and *A. niger* (molds).^47^

#### 2.3.2 Starch and thermoplastic starch (TPS)

Starch is a polysaccharide, which consists of glucose units and two branches (amylopectin and linear amylase). Furthermore, starch is consider as a widely accessible polysaccharide, which is obtained through different types of crops.^48^ In addition, it is considered as a promising alternative material for the fabrication of biodegradable materials instead of the currently used synthetic polymers.^19^ Starch is utilized in different food and non-food applications, especially in agriculture, medicine, and packaging.^49^ The blending of starch with any synthetic or natural polymer^50^ or the incorporation of nanoparticles into starch^51^ are promising pathways to enhance novel biodegradable materials with amended features. In addition, another approach has been studied for the utilization of starch, which has been chemically adapted *via* oxidation or carboxymethylation.^52^ Starch is extensively studied as a material for food packaging because it has several benefits such as its availability, non-toxicity, biodegradability, in addition to its stability in air. Furthermore, native starch granules can be subjected to hydrolysis for a long time below the gelatinization temperature; here, the non-crystalline region is hydrolyzed and leads to the separation of the crystalline lamellae, which is highly resistant to hydrolysis. It has been observed that the positively charged ion that is present on the surface of the antimicrobial agent contributes in its antimicrobial action. Accordingly, the antimicrobial effect of the incorporated/adsorbed metals on the polysaccharides' surface increase the exposed surface area.^33^

• TPS with silver nanoparticles

The most successful and economically viable component of the group of biodegradable polymers is starch. This material has several benefits, including wide applicability and simplicity of use, complete biodegradability without the formation of poisonous residues, and low cost, which enhances the biodegradability of non-biodegradable plastics when mixed with them. Despite its various benefits, starch alone is not a great choice for packaging due to its bad mechanical characteristics, powerful hydrophilic property, and crystalline nature, which result in bad processing capacity. To apply starch as a food packing material that shows thermoplastic properties, it is necessary to add glycerol in addition to low molecular weight polyhydroxy compounds, such as urea and polyethers. These plasticizers improve the process-ability as well as the flexibility by reducing the intramolecular hydrogen bonding of the starch film. TPS films still need additional properties such as good mechanical properties and lower sensitivity to moisture for using in food packaging applications. To overcome this problem, a nanocomposite film of silver nanoparticles with starch was prepared and the mechanical, gas barrier, and antibacterial properties were investigated, in addition to its safety of use, because the migration of silver from the nano-film was discovered to be within the permissible limit.^53^

• TPS with talc nanoparticles

**Fig. 7:**
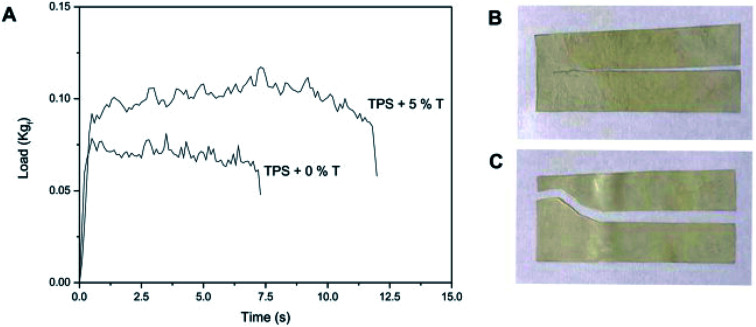
(A) Load–time curves of the films based on thermoplastic corn starch (TPS) with 0 and 5% w/w talc nanoparticles obtained from the propagation tear resistance assays. The tested specimens of TPS films with (B) 0 and (C) 5% w/w talc nanoparticles. Reproduced with permission from ref. 46 Copyright© 2017 Elsevier.

In a recent work, authors discussed the variation in the properties of the prepared film with different concentrations of the added talc; they found that the film barrier characteristics were identified for measuring the tightness of the packaging bags. By adding talc (3% w/w) to thermoplastic starch, water vapor and oxygen permeability decreased by 54% and 26%, respectively. Talc is a layered phyllosilicate consisting of [Mg(OH)_2_] sheets of octahedral brucine inserted into two sheets of tetrahedral silica [Si_2_O_5_]. In addition, talc is collected from two types of surfaces: an inadequately energetic “basal surface” with basic and hydrophobic Si–O–Si groups in addition to a more energetic “edge” with acidic and hydrophilic Si–OH groups and residual magnesium cations. The film stiffness revealed that low talc concentration has no significant effect. On the other hand, 3% w/w of talc increased the stiffness by 15%, while 5% w/w of talc increased the Young's modulus up to 68% and 81%, respectively, in case of tensile and quasi-static investigations. It was found that the water vapor permeability (WVP) of the film without the nanoparticles was greater due to the hydrophilic nature of starch. Alternatively, 1% w/w of talc did not cause a significant change in the WVP of the TPS films but a significant decrease in the WVP by about 1.4 times was recorded for 3% w/w of talc. Accordingly, talc nanoparticles act as an obstacle in preventing water vapor from passing through the film. Normalized load required to propagate tear across starch films without talc particles resulted 0.31 ± 0.02 kgf mm^−1^ while for TPS bionanocomposites with 5% w/w talc was 0.44 ± 0.02 kgf mm^−1^ (Fig. 7).^54^

#### 2.3.3 Semolina with imbedded nanokaolin

Semolina flour is a type of wheat that includes high level of gluten and modifies the dietary characteristics of foodstuffs among multiple flours. Semolina is a light-colored grain that is extra-hard, transparent, and shows antioxidant activity. The good characteristics of semolina for use as an edible film and the potential for strengthening its mechanical, physicochemical, and barrier characteristics by nanokaolin-enhanced semolina have been reported. Nanokaoline improved several characteristics such as water susceptibility, water vapor permeability (WVP), permeability to oxygen, mechanical, and barrier properties. For, for water susceptibility, using a huge amount of nano-kaolin was associated with a gradual reduction in the moisture content in the film. The interaction between the plasticizers, biopolymers, and nano-kaolin lowered the interaction of the hydroxyl group with water, resulting in a less hydrophilic matrix that is probably responsible for this property. Secondly, the water vapor permeability (WVP), which is defined as the moisture transfer from the ambient air to food or between two parts of a heterogeneous product of different humidity values, was 8.61 × 10^−7^ but this value can be lowered by raising the concentration of kaolin. For example, adding 5% of kaolin can decrease the WVP to 4.58 × 10^−7^. The addition of nanoparticles to the film path will lower the oxygen permeability coefficient because it creates convoluted path for the oxygen molecule to travel through. As result of this, the oxygen permeability is lowered by raising the kaolin content in the matrix. The mechanical features such as the tensile strength (TS), elongation-at-break (EB), and Young's modulus (YM) also increase on adding kaolin nanoparticles to the matrix.^55^

#### 2.3.4 Cellulose

Cellulose (β-1,4-d-glucopyranose polymer) is considered as the biopolymer that comes from bacteria, algae, and higher plants.^56^ It has the select ability to preserve water loss from dry areas and at the same time, it can absorb undesirable liquids from a wound. This property accelerates the healing of deep ulcers. However, this property of cellulose raises the chance of microbial development; accordingly, antimicrobial agents with cellulose can be utilized in wound dressings. Cellulose is additionally used as a supporting substance for some nanomaterials that can expand the exposed surface of the nanoparticles, leading to their enhanced action. Furthermore, adsorbed silver nanoparticles on cellulose fibers, through the interactions of oxygen (from cellulose) with silver, display great antimicrobial activity up to 99.99% against *S. aureus* and *E. coli*.^57^ In a recent work, silver, gold, and platinum nanoparticles were synthesized and enforced in a cellulose gel *via* the hydrothermal reduction technique, followed by drying using supercritical CO_2_. The produced aerogels had unique features such as extraordinary porosity, surface area, transmittance, mechanical strength, and moderate thermal stability.^58^ Previous research has investigated the properties of the prepared nanocellulose material through the crystallinity index, which decreased in contrast to microcrystalline cellulose, thus raising the probability of using it as an enhancing agent in the preparation of biodegradable composite films.^59^ Another previous work has shown that the amalgamation of cellulose nanocrystals inside starch-based nanocomposite films leads to the control of d-limonene permeability.^60^ In addition, cellulose was used in order to confirm the antibacterial capacity (*E. coli* and *S. aureus*).^61^

### 2.4 Biodegradability of common imbedded nanoparticles

#### 2.4.1 Ag NPs

Advanced studies have improved the properties of biodegradable food packaging materials by using silver nanoparticles (AgNPs). A recent review has intensively discussed the safety issue of the migration level of AgNPs. AgNPs are considered safe when their migration level in food is below the maximum migration limit that is specified by the European Union (EU) and USA food safety authorities. Silver cation is used to evaluate the migration level into the packed food. The recommendations are not to exceed 0.05 mg L^−1^ in water medium and 0.05 mg kg^−1^ in food.^62^ A comprehensive study was performed on the migration of Ag ions or particles from several types of nanomaterials such as LDPE and polypropylene into food. Their results showed that acidic food and the classical oven conditions led to the highest migration level.^63^

A nanocomposite film consisting of chitosan, gelatin, and polyethylene glycol as the host materials and silver nanoparticles was investigated using the solution casting method. AgNPs were embedded to improve the mechanical properties and to reduce the visible light penetration. This study reported that film was appropriate as an antimicrobial, biodegradable food packaging material.^64^

One of the most common clay minerals is montmorillonite K10 (MMT-10K), which is considered biocompatible and biodegradable, and has good mechanical properties. Furthermore, AgNPs are used due to their effective antimicrobial and pathogenic activity. A recent study developed a biodegradable polyvinyl alcohol (synthetic polymer)/clay/silver nanocomposite as a novel packaging pouch for chicken sausage. It was prepared *via* an eco-friendly technique (using ginger extract for the *in situ* generation of silver nanoparticles). This study deduced that the nanocomposite clay film (PAGM) was fully degraded within 110 days. It took a longer time compared to the PVA/ginger extract (PG), PVA/MMT (PM), and PVA/ginger extract/MMT (PGM) films due to the presence of AgNPs. Although the nanocomposite PAGM film took more time to degrade compared with the other types, it degraded faster than the native PVA (P) film in addition to its effective antimicrobial and antipathogenic properties.^65^

#### 2.4.2 ZnO NPs

Advanced studies have developed enhanced biodegradable food packaging materials by using zinc oxide nanoparticles, which is safe because of its low toxicity and chemically inactivity.^66^ It is safe as it is listed under “Generally Recognized As Safe” (GRAS) materials (21CFR18228991) by the United States Food and Drug Administration (USFDA) in 2014; thus it can be utilized in cereal-based food protection.^67^ The synthesized biodegradable film composed of soybean protein isolate (SPI) as the host material and zinc oxide as the nanoparticles improved the antimicrobial, thermal barrier, and mechanical properties.^66^ Another work investigated biodegradable nanocomposite materials using solution casting method including starch, amino acid (lysine), and polypropylene glycol (PPG) as the host materials and ZnO as the nanofiller.^68^ The nanocomposites were fabricated with different concentration of ZnO nanoparticles, for instance, 0 wt%, 1 wt%, 3 wt%, and 5 wt% by keeping the peptide content constant in addition to changing the ratio between the starch and ZnO nanoparticles concentration. They found that greater the content of ZnO NPs, the better the mechanical properties of the resulting material. Moreover, the solubility of the starch/lysine films without ZnO NPs was 100%, while the addition of 1% ZnO NPs reduced the solubility to 43%. By increasing the concentration of ZnO NPs to 3%, the solubility again decreased to 38% but the further addition of ZnO NPs (5%) to the film caused only a slight change by reducing the solubility to 37%. All of the obtained properties can enhance the biodegradability of the nanocomposite films in several biological applications.

A previous research study has reported the synthesis of biodegradable food packaging materials from mahua oil-based polyurethane (PU) and chitosan (CS) as the host materials, and different quantities of zinc oxide nanoparticles were included as the nanofiller.^69^ The biodegradation degree of the film was ascertained by recording the weight loss at different time intervals. In addition, the obtained results indicate the existence of outstanding UV screening aptitude, great transparency, and a high level of biodegradability up to 86% in 28 days. In addition, the biodegradable PU/CS/5% ZnO NPs film is promising as a biodegradable food packaging material.

#### 2.4.3 MgO NPs

Magnesium oxide nanoparticles (MgO NPs) were found to increase the features of biodegradable food packaging materials as well as their antibacterial properties.^70^ Biodegradable rice starch (RS) film was investigated as the host material and embedded magnesium oxide as the nanoparticles. The RS/MgO NPs films were used as biodegradable food packaging materials with good antibacterial properties. Furthermore, MgO is listed as one of the generally recognized as safe (GRAS) compounds by the US Food and Drug Administration (FDA).^67^

#### 2.4.4 Zein nanoparticles

Zein nanoparticles (Z Nps) are a safe (GRAS) biomaterial and have properties such as low toxicity, biodegradability, and biocompatibility; thus, more studies should focus on it to manufacture biodegradable and environment-friendly food packaging materials.^71^ A protein-isolated (WPI) nanocomposite biodegradable film was studied as the host material and zein as the nanoparticles. Z Nps was used to develop the mechanical properties and the water vapor barrier of WPI without negatively affecting the elongation of the films. The WPI/Z NPs nanocomposite films are highly suitable for use as biodegradable food packaging materials.^71^

#### 2.4.5 Magnetite nanoparticles (Fe_3_O_4_)

Magnetite nanoparticles (MNPs) were studied for their use in food packaging materials by utilizing them as a nanofiller in dialdehyde starch (DAS) due to the availability and non-toxicity of starch. Several works that report the studies related to magnetite nanoparticles as fillers in starch materials and their applications in food packaging have proven that the MNPs/DAS composite film is a potential candidate with better characteristics (low moisture content).^72^

#### 2.4.6 SiO_2_ nanoparticles

**Fig. 8:**
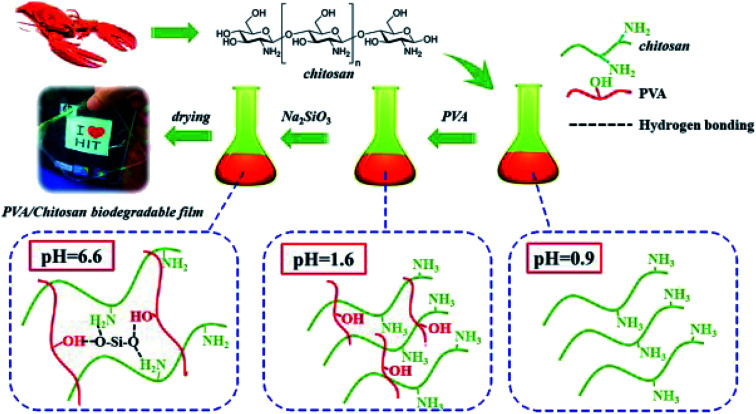
SiO_2_*in situ* enhanced the PVA/CS biodegradable films by hydrolysis of sodium metasilicate. Reproduced with permission from ref. 64 Copyright© 2018 Elsevier.

Yu *et al.* created a biodegradable composite film encompassing silica, PVA, and chitosan (Fig. 8).^73^ In addition, the obtained film can be used in food packaging owing to its biodegradable, low cost, and high performance properties. For the film with 0.6 wt% SiO_2_, the tensile strength of PVA/chitosan was as high as 44.12 MPa and increased by about 45% through hydrogen bonding between silica and PVA or chitosan. SiO_2_ also decreased the moisture and oxygen permeability of the food packaging films to maintain the freshness. SiO_2_ nanomaterials are considered as suitable compounds that are generally recognized as safe (GRAS) by the US Food and Drug Administration (FDA);^67^ thus, it is possible to use them in human and animal food industries. The composite film is valuable and is favorable as a future green, high-performance, and environment-friendly food packaging. In addition, Table 1 shows the biodegradability of different commonly imbedded nanoparticles, confirming the functionality of these nanoparticles with the possible matrix formed as well as the applicable findings attained through these materials.

**Table 1:** Biodegradability of commonly imbedded nanoparticles showing the functionality of these nanoparticles with the possible matrix formed as well as the applicable findings attained through these materials

Imbedded nanoparticles	Functionality	Matrix	Findings	Ref.
Ag NPs	To improve the mechanical properties	Chitosan, gelatin, polyethylene glycol	The film was appropriate as an antimicrobial biodegradable food packaging material	64
	To reduce visible light penetration			
	They are used for their effective antimicrobial and pathogenic activity	Montmorillonite K10, polyvinyl alcohol (PVA)	The nanocomposite film was fully degraded within 110 days	65
			It had effective antimicrobial and antipathogenic activities	
ZnO NPs	To enhance the biodegradable food packaging materials	Soybean protein isolate (SPI)	It improved the antimicrobial, thermal barrier, and mechanical properties	74
	Nanofiller	Starch, amino acid (lysine), polypropylene glycol (PPG)	The higher the ZnO NPs content, the better the mechanical properties and lower the solubility of the resulting material	68
	Nanofiller	Mahua oil-based polyurethane (PU) and chitosan (CS)	Biodegradability depends on humidity and the chemical structure	69
			The presence of ZnO NPs supports the film's hydrophobicity by about 63%	
			Excellent UV screening ability	
			High transparency	
			High degree of biodegradation up to 86% in 28 days	
MgO NPs	To improve the properties of biodegradable food packaging materials and their antibacterial properties	Rice starch (RS)	It can be used as a biodegradable food packaging material with good antibacterial properties	70
Zein NPs	To improve the mechanical properties and the water vapor barrier of the protein isolate without negatively affecting the elongation of the films	Protein isolate	The nanocomposite films were suitable to be used as biodegradable food packaging materials	71
Magnetite NPs (Fe_3_O_4)_	Nanofiller	Dialdehyde starch	It was considered as a potential candidate with better characteristics (low moisture content)	72
Silica (SiO_2_)	To increase the tensile strength through hydrogen bonding between silica and PVA or chitosan	PVA/chitosan	Decreased the moisture and oxygen permeability of the food packaging films to maintain the freshness	73
			Increased the tensile strength	

### 2.5 Possibility of migration of different nanoparticles (NPs) into foodstuffs

The mass transfer process through low molecular mass compounds in packaging materials is known as the migration of materials into foodstuffs, where different amounts of these substances can be release inside the contained food product.^75^ In the last few years, many research studies have been carried out in order to check the possibility of migration of harmful contents of the utilized materials in food packaging towards the food content and to improve the methods of fabrication of novel food packaging contact materials in accordance with the specific migration tests and the limits of materials either in food or inside the food simulant, as prescribed by the European regulation.^76,77^ Furthermore, the detected food simulant limits for six types of substances are as follows: ethanol 10% v/v; acetic acid 3% v/v; ethanol 20% v/v; ethanol 50% v/v; vegetable oil; and poly(2,6-diphenyl-*p*-phenylene oxide), and all of them are related to the migration of the packing materials towards the food content. Nanoparticles (NPs) and nanomaterials are considered as appropriate materials for the food packaging and since there are not many previous studies related to them, more research work is needed so as to aid their proper utilization.^78^ Furthermore, the studied nanoparticles, especially in the composites, illustrate the complications in characterization and analysis during the migration studies.^79^ In addition, several research studies have been undertaken to check and characterize the nanoparticle materials *via* different methodologies and strategies.^80,81^ After that, the researchers predicted the migration of nanoparticles through physical-chemical models and established the low and slow rate of migration of NPs from the food packaging material to the foodstuffs.^82^ In addition, another study indicates that when the viscosity of the nanoparticle/polymer composite decreases, the migration rate of the system increases.^83^ One of the pioneering studies concerning the migration of NPs inside the foodstuff examined the migration of nanoclay particles inside vegetables packaged with biodegradable starch/clay nanocomposite films.^84^ Nevertheless, depending on the European regulation, the obtained results, using the simulant of fatty foods (ethanol 95%), indicated that the utilized materials have less migration and can be used safely in the food packaging sector.^85^ The inclusive migration of the tested treatments (with the maximum value of 8 mg dm^−2^) was much below the entire prescribed migration limit (10 mg dm^−2^) recognized for the materials.^85^ In conclusion, in order to understand the mechanism of the diffusion process during migration as well as the crystalline structure of the NP materials utilized for food packaging through size and morphology characterization for assessing the risk to human health upon consumption/exposure, further research is recommended.

### 2.6 Antimicrobial activity of biodegradable packaging materials

Recently, biodegradable materials are the most commonly used materials in food packaging and with the progress of biodegradable antimicrobial packaging, which have become the most suitable materials as they are green, reproducible, and environment-friendly, for example, polylactic acid (PLA), cellulose, starch, chitosan, and gelatin.^86,100^ In addition, Table 2 illustrates the antimicrobial activity of different biodegradable packaging materials with several additives and at different concentrations.

**Table 2:** Antimicrobial activity of different biodegradable packaging materials with several additives and concentrations

Types of nano-packaging material	Additives	Concentration	Antimicrobial effect	Ref.
PLA	*Mentha piperita* essential oil (MPO), *Bunium percicum* essential oil (BPO), nanocellulose (NC)	1% (w/v) PLA, 0.5% (v/v) MPO, 1% (v/v) NC	The film has antimicrobial activity against *Staphylococcus aureus*, Enterobacteriaceae, and *Pseudomonas*	88
			The inoculated bacteria in the cell concentration are about 1 to 10^7^ CFU mL^−1^	
	Quince seed mucilage (QSM) films with *Origanum vulgare* L. *virens* essential oil (OEO)	1% OEO	The film prevents the growth of *S. aureus* and *E. coli* but it has no effect on *Salmonella typhimurium* and *Pseudomonas aeruginosa*	89
			The density was then set to 0.5 for 250 MacFarland (approx. 10 CFU mL^−1^) where the agar diffusion method was used to estimate the antibacterial activity	
Starch	Potassium sorbate	0.3% w/w potassium sorbate	The film inhibits the growth of *Candida* spp., *Penicillium* spp., *S. aureus*, and *Salmonella* spp	92
			Starch films with diameters of 1 and 3 cm were cut at 10^8^–10^9^ and 10^7^–10^8^ CFU mL^−1^ correspondingly, and the method used to determine the antimicrobial activity was agar diffusion	
	Lauric acid and chitosan	8% lauric acid was added based on the percentage of starch, starch and chitosan with different mixing ratios	The film inhibits *B. subtilis* more than *E. coli* in solid or liquid media, so the multicomponent film has better effect on *B. subtilis*	93
	Thyme essential oil (TEO)	*% (v/v)	*E. coli* and *S. Typhi* reduced within 5 days	94
			The antimicrobial effect for the film was verified by agar diffusion method where the plates were spiked with 0.1 mL of inoculum containing bacteria with 10^5^ CFU mL^−1^	
Chitosan (Ch)	Cinnamon essential oil (EO)	0.4%, 0.8%, 1.5%, and 2% (v/v)	The antimicrobial activity was increased	96
			Agar diffusion method was used where it was noticed that after 24 hours of incubation, the media had a bacterial count of more than 1 × 10^9^ CFU mL^−1^	
	Polyvinyl alcohol (PVA) containing mint extract (ME)/pomegranate peel extract (PE)	1% Ch, 5% PVA, 1% ME, 1% PE	It showed antibacterial activity against *S. aureus* and *Bacillus cereus*	97
			About 10^4^–10^5^ of the bacterial concentration of the cells were incubated for 24 hours	
	Green tea extract	20% (w/v)	Effectively inhibits the microbial growth (total aerobic counts, yeasts molds, and lactic acid bacteria) at 4 °C	98
	*S*-Nitroso-*N*-acetyl-d-penicillamine (SNAP)	2 wt%	It inhibits *E. faecalis*, *S. aureus* and *L. monocytogenes*	105
Cellulose	Nisin	2500 IU mL^−1^	Inhibits the growth of *L. monocytogenes* after 14 days of storage	100
			It was determined by diffusion assay	
			It displays antimicrobial properties against several Gram-positive bacteria (*Bacillus subtilis* and *Staphylococcus aureus*)	101
Gelatin	Oregano (*Origanum vulgare*), rosemary (*Rosmarinus officinalis*), leaves of murta (*Ugni molinae*)		Increased the antioxidant capacity and antimicrobial activity towards fish-derived gelatin films, which was measured *via* viable cell count method	104
	Thyme essential oil (TEO) in skate skin gelatin (SSG) film	1%	The film inhibits the growth of *L. monocytogenes* and *E. coli* in the packaging of chicken tenderloin samples	106

#### 2.6.1 Polylactic acid (PLA)

Polylactic acid (PLA) derived from renewable and biodegradable resources such as corn, which has ideal chemical and physical properties because its biocompatibility, biodegradability, renewability, non-toxicity, hydrophilicity, water solubility, and compostable and hydrophobic surface properties, is usually added to several polymers, which make it one of the most unique and promising materials in food packaging applications.^87^

Talebi *et al.*^88^ prepared antimicrobial films by combining various concentrations of *Mentha piperita* essential oil (MPO), *Bunium percicum* essential oil (BPO), and nanocellulose (NC) into PLA films, stored the films at 4 °C for 12 days, and then measured the antimicrobial effect to show their antimicrobial activity against *Staphylococcus aureus*, Enterobacteriaceae, and *Pseudomonas*. The water vapor barrier property of the prepared film was improved by adding *Bunium percicum* EO (BPO).^88^ The antibacterial activity of the film consisting of polylactic acid (PLA) containing cellulose nanoparticles, *Bunium persicum*, and *Mentha piperita* essential oils (EOs) was found to be about 1 to 10^7^ CFU mL^−1^. In addition, M. Jouki *et al.*^89^ examined new active packaging films that show antimicrobial properties against yeasts and molds.^77^ The produced films with 5% and 10% of OEO are the most effective and show appropriate mechanical and physical features with small modifications. Also, by the addition of quince seed mucilage (QSM) films with OEO, the produced films show antibacterial properties and the film with 1% OEO prevents the growth of *S. aureus* and *E. coli* but it had no influence on *Salmonella typhimurium* and *Pseudomonas aeruginosa*. The inoculum density was then set to 0.5 for 250 MacFarland (approx. 10 CFU mL^−1^). The agar diffusion process was utilized for estimating the antibacterial activity.

#### 2.6.2 Starch

Starch is the cheapest of the group of biodegradable polysaccharides. Several sources of starch such as potato, cassava, rice, corn, and tapioca have been used for the production of biopolymers.^90^ Starch acts as a thickener and an additive, so it considered as a promising candidate for food packaging applications. It considered as a moderate oil barrier. It has hydrophilic function groups in its molecular structure and so it is not applicable in a humid environment.^91^

Starch-based films are suitable as antimicrobial packaging materials. Lopez and Olivia studied various starch film formulations prepared with potassium sorbate (0.3% w/w) at different pH levels.^92^ Starch films with diameters of 1 and 3 cm were cut at 10^8^–10^9^ and 10^7^–10^8^ CFU mL^−1^, respectively, and the antimicrobial activity was determined by the agar diffusion method.

The results show that a change in the source and pH of starch has no effect on the kinetic release. Furthermore, Salleh *et al.*^93^ utilized lauric acid (8%), which was added based on the percentage of starch (starch and chitosan with different mixing ratios), and chitosan as the antimicrobial agents to fabricate an antimicrobial packaging from wheat starch.^92^ The antimicrobial properties were confirmed against *B. subtilis* and *E. coli*; the obtained results indicate that these materials are good as food packaging materials and can keep food fresh and free from bacterial contamination.

Issa *et al.*^94^ developed a biodegradable sweet potato starch/montmorillonite (MMT) film for food packaging, which was stimulated with thyme essential oil (TEO) (6%, v/v). The mechanical properties of starch films could be efficiently enhanced by adding MMT. They found that the *E. coli* and *S. Typhi* (*p* < 0.05) colony forming units reduced to measurable points afterward EOs within 5 days, while the control group, devoid of EOs, had almost 4.5 log colony forming units (CFU)/g. The antimicrobial effect of the film was verified by the agar diffusion method, where the plates were spiked with 0.1 mL of inoculum containing bacteria with 10^5^ CFU mL^−1^.

#### 2.6.3 Chitosan

Chitosan films have fascinating features that enable their utilization as environment-friendly food packaging materials.^95^ Chitosan acts as an antimicrobial agent and a polymer substrate. Ojagh *et al.*^96^ fabricated a high-performance biodegradable film by mixing two antimicrobial agents with chitosan and variable concentrations of cinnamon essential oil (EO), such as 0.4%, 0.8%, 1.5%, and 2% (v/v). The film showed low affinity towards water and the antimicrobial activity was increased. The agar diffusion method was used, where it was noticed that after 24 hours of incubation, the media had a bacterial count of more than 1 × 10^9^ CFU mL^−1^.

Kanatt *et al.*^97^ prepared good antioxidant composite films by incorporating polyvinyl alcohol (PVA) containing mint extract (ME)/pomegranate peel extract (PE) into chitosan, 1% Ch, 5% PVA, 1% ME, and 1% PE. The peel extracts enhanced the tensile strength of the films (41.07–0.88 MPa) without disturbing their puncture strength. In addition, the films were found to have antibacterial activity against *S. aureus* and *Bacillus cereus*.^96^ Also, about 10^4^–10^5^ of the bacterial concentrations of the cells were incubated for 24 hours at a temperature of about 37 °C.

Chitosan has a decent film-forming and developing ability and can be easily utilized with other bioactive agents, which can together be utilized as environment-friendly food packaging materials and can provide chitosan with various promising properties.^95^ Chitosan has also been used an antimicrobial agent and a polymer substrate. In addition, the film showed low affinity towards water and the antimicrobial activity was increased owing to the contact effect between cinnamon EO components and chitosan.

In another work,^98^ active packaging for pork sausages by the combination of green tea extract (20%, w/v) and a chitosan film was developed. The film effectively inhibits microbial growth at a temperature of 4 °C, had lower variability of total aerobic counts, yeasts molds, and lactic acid bacteria, showed improved antioxidant as well as antimicrobial features, and enhanced the food protection ability. Furthermore, chitosan films with 0, 2.5%, 5%, 10%, and 20% (w/w) propolis extract have also been established. Besides, polyphenols are considered as the dominant molecules in propolis extract. In addition, the mechanical properties and the antioxidant activity of the film were significantly improved. Some works have suggested that some connection between the chitosan and propolis extract can improve the antimicrobial features of the films.^96^

#### 2.6.4 Cellulose

Cellulose is the most environment-friendly and biodegradable raw material, which is obtained from living organisms and is a linear homopolysaccharide.^86,99^ Nguyen *et al.* fabricated a green and high-performance cellulose film for meat packaging applications consisting of nisin (2500 IU mL^−1^) and cellulose.^100^ The prepared film inhibits the growth of *L. monocytogenes* after 14 days of storage *via* the diffusion assay. The result indicates that the addition of nisin improved the antimicrobial properties. Saini *et al.* established appropriate cellulose fiber-based nisin for use as an antimicrobial food packaging.^101^ In addition, the produced film displays antimicrobial features against several Gram-positive bacteria. Mixing nisin with cellulose nanofibers can destroy *B. subtilis*.

Sundaram *et al.*^102^ organized chitosan nanocellulose films with the antimicrobial material (2 wt%) *S*-nitroso-*N*-acetyl-d-penicillamine (SNAP), which caused a change in the level of nitric oxide (NO) released that caused a zone of inhibition (ZOI) (mm) with different diameters, which was depicted the level of NO release. The antimicrobial activity of the membranes caused a similar ZOI between 2-layer and 3-layer membranes against *E. faecalis* and *S. aureus*. However, *L. monocytogenes* displayed a significant variance in the ZOI between the 2-layer and 3-layer membranes. *L. monocytogenes* was most susceptible bacteria to both the 3-layer and 2-layer membranes.

#### 2.6.5 Gelatin

A previous study by Nur Hananie *et al.*^103^ stated that gelatin has antioxidant and antimicrobial activities. However, the antimicrobial activity is due to peptide characteristics. The researchers observed that gelatin is a carrier of bioactive contaminents.^104^ Oregano (*Origanum vulgare*), rosemary (*Rosmarinus officinalis*), and leaves of murta (*Ugni molinae*) were utilized as natural antioxidants and/or antimicrobial materials to enhance the antioxidant capacity towards fish-derived gelatin films (measured *via* viable cell count method) as well as to encompass the features of these biodegradable films and produce an active packaging biomaterial.^104,107–114^

## 3 Conclusion and future perspectives

In recent years, different biological applications have become highly developed by using smart eco-friendly nanomaterials or nanocomposites. It is very promising that nanotechnology is being used in food packaging because this technique could enhance food protection and superiority while decreasing the utilizing of precious raw materials and waste generation. Most new features of food packaging products will probably be attributed to nanomaterials, either directly or indirectly, because they are necessary for improving gas-barrier, antimicrobial, mechanical, and thermal properties of the packaging materials. Nanomaterials play the important role of developing several properties of biodegradable food packaging materials such as mechanical properties, water or gas barriers, and antimicrobial activity, thus leading to the increased shelf-life of stored food by the prevention of spoilage to some extent. On the other hand, replacing non-biodegradable materials such as polyethylene by other biodegradable materials will save our environment as well as our health. But it is important to note that, till now, there is/are no discovered biodegradable food packaging materials that can be a completely good alternative to traditional ones. Therefore, further studies on improving the properties of biodegradable food packaging materials are still needed.

## Conflicts of interest

The authors declare no conflict of interest.

## Supplementary Material
